# Potential probiotic approaches to control *Legionella* in engineered aquatic ecosystems

**DOI:** 10.1093/femsec/fiac071

**Published:** 2022-06-09

**Authors:** Alessio Cavallaro, William J Rhoads, Simona G Huwiler, Elyse Stachler, Frederik Hammes

**Affiliations:** Department of Environmental Microbiology, Eawag, Swiss Federal Institute of Aquatic Science and Technology, 8600 Dübendorf, Switzerland; Department of Environmental Systems Science, Institute of Biogeochemistry and Pollutant Dynamics, ETH Zurich, 8092 Zurich, Switzerland; Department of Environmental Microbiology, Eawag, Swiss Federal Institute of Aquatic Science and Technology, 8600 Dübendorf, Switzerland; Department of Plant and Microbial Biology, University of Zurich, 8008 Zurich, Switzerland; Department of Environmental Microbiology, Eawag, Swiss Federal Institute of Aquatic Science and Technology, 8600 Dübendorf, Switzerland; Department of Environmental Microbiology, Eawag, Swiss Federal Institute of Aquatic Science and Technology, 8600 Dübendorf, Switzerland

**Keywords:** *Legionella*, probiotics, antagonism, competition, biofilm, protozoa, pathogen–host interaction, predation

## Abstract

Opportunistic pathogens belonging to the genus *Legionella* are among the most reported waterborne-associated pathogens in industrialized countries. *Legionella* colonize a variety of engineered aquatic ecosystems and persist in biofilms where they interact with a multitude of other resident microorganisms. In this review, we assess how some of these interactions could be used to develop a biological-driven “probiotic” control approach against *Legionella*. We focus on: (i) mechanisms limiting the ability of *Legionella* to establish and replicate within some of their natural protozoan hosts; (ii) exploitative and interference competitive interactions between *Legionella* and other microorganisms; and (iii) the potential of predatory bacteria and phages against *Legionella*. This field is still emergent, and we therefore specifically highlight research for future investigations, and propose perspectives on the feasibility and public acceptance of a potential probiotic approach.

## Teaser

Here we consider a variety of probiotic strategies against *Legionella*, focussing on the potential and feasibility, the main research gaps, and public acceptance.

## Introduction

Several species within the genus *Legionella* are opportunistic human pathogens that act as the etiological agents of Legionellosis, which manifests as either Legionnaires’ disease, a severe pneumonia, or Pontiac fever, a mild flu-like illness (Fields et al.^2002^). *Legionella* have been detected in a variety of engineered aquatic ecosystems including wastewater treatment plants, cooling towers and drinking water systems (Caicedo et al. 2019, Falkinham et al. 2015). In drinking water, *Legionella* are found more often, and at higher concentrations, in building plumbing systems (Falkinham et al. 2015), where favourable environmental conditions for growth include a high pipe surface area, warm water temperatures, high water retention times, low or no secondary disinfectant residuals, and additional nutrients migrating from plumbing components (Falkinham et al. 2015, Proctor et al. 2016, Rhoads et al.^2016^).

**Figure 1.**
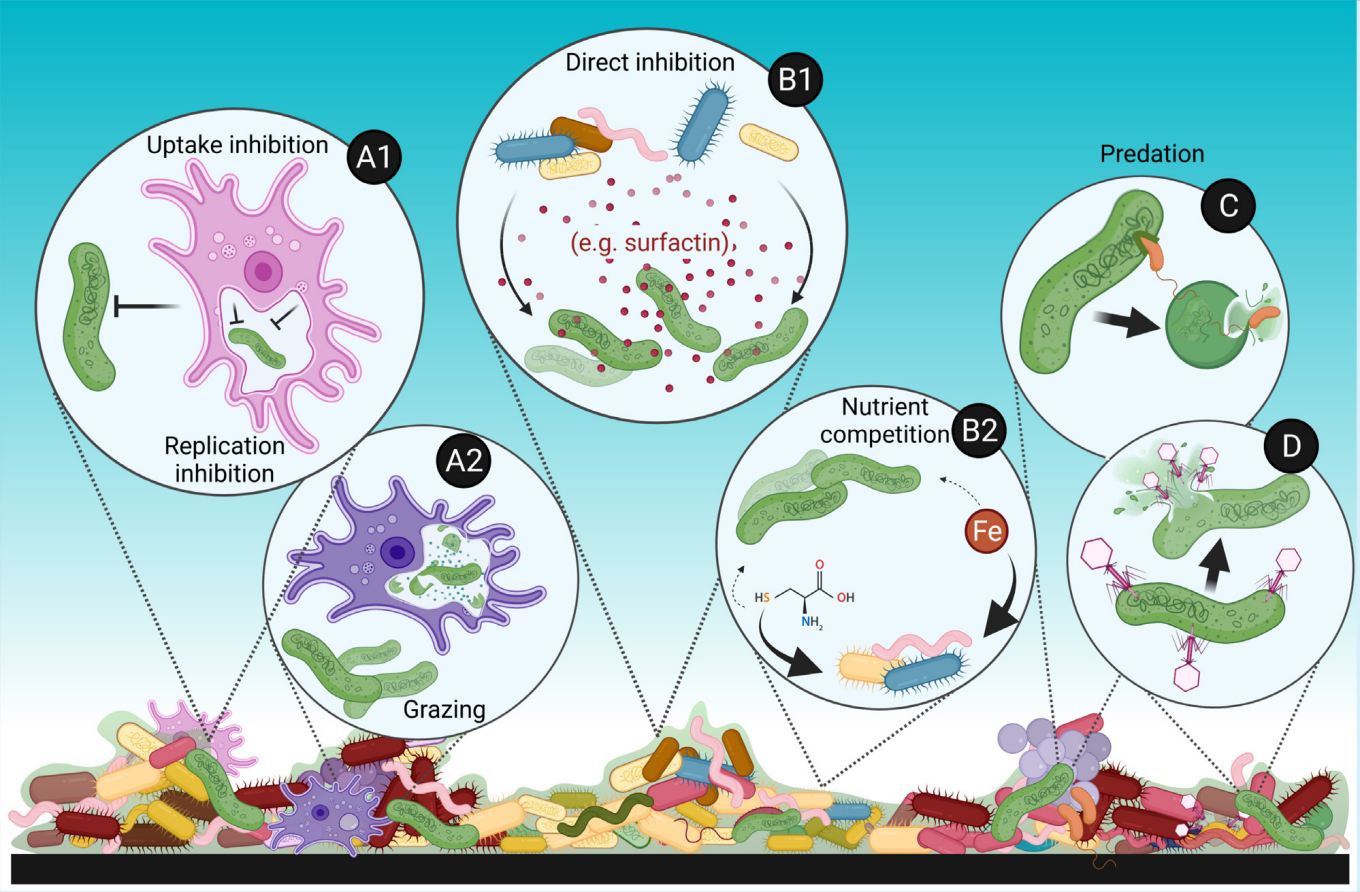
Overview of ecological interactions of *Legionella* (shown in green) in a complex biofilm community that could be potentially exploited to design targeted probiotic strategies against pathogenic *Legionella* species in engineered aquatic ecosystems. **(A1)** Uptake/replication inhibition of *Legionella* caused by other symbionts of their protozoan hosts (Section 2); **(A2)** Grazing of *Legionella* by specific protozoan hosts (Section 2); **(B1, B2)** Interference and exploitative competition with other bacteria (Section 3); **(C-D)** Predation by predatory bacteria (brown) and phages (violet) (Section 4). Created with Biorender.com.

However, *Legionella* do not exist in isolation. These bacteria are members of the complex microbial communities found in drinking water systems, and interactions with other microorganisms can sometimes promote or inhibit their growth (Figure 1). For example, *Legionella* proliferate within protozoan hosts (e.g., *Acanthamoeba* spp.), exploiting their intracellular environment to replicate and gain protection against external stressors (Declerck, 2010, Taylor et al.^2009^). Also, several laboratory-scale studies have shown bacterial isolates that directly benefit or inhibit *Legionella* growth on pure culture agar plates (Corre et al. 2018; Paranjape et al. 2020).

Physical and chemical engineering controls for *Legionella* sometimes fail for reasons related to underlying microbial physiology. For instance, *Legionella* can survive and adapt to hot water temperatures when superheating cycles are applied to a given system (Allegra et al. 2011). Also, the fact that *Legionella* live predominantly embedded in biofilms and grows intracellularly inside of eukaryotic hosts, protects them from chemical disinfection (Boamah et al. 2017, Mondino et al. 2020, Winiecka-Krusnell and Linder 1999). Hence, the opportunity exists for alternative or supplementary interventions that incorporate the interactions of *Legionella* with the surrounding microbial community across multiple trophic levels. Wang and colleagues (Wang et al.^2013^) explored the idea of a “*probiotic*” approach to control opportunistic pathogens and hypothesised that maintaining a desirable plumbing microbiome by manipulating physical/chemical characteristics, taking advantage of competitive ecological niches, encouraging growth of antagonists, and/or eliminating keystone species, could exclude the colonisation and growth of pathogens. Given the development of new molecular analysis tool sets, the increase in annotation libraries and multiple fundamental or observational studies reported during the last decade, the field is ripe for an update and extension of this concept.

In this review, we expand on the work of Wang and colleagues (Wang et al. 2013), focusing specifically on control of *Legionella* in three key areas: (i) disrupting the protozoan-host replication cycle, (ii) antagonistic interactions with other bacteria, and (iii) predation by predatory bacteria or phages. In the context of engineered aquatic ecosystems such as building plumbing systems, and for the purpose of this review, we define a “probiotic” as the purposeful addition of harmless microorganisms that modulate the microbial composition of the system to inhibit or exclude pathogens. In this framework, a number of different ecological processes and mechanisms will be explored for their potential to contribute to future probiotic strategies, while the practical implication and remaining research gaps of such approaches are considered as well.

## *Legionella*–protozoa interactions

### Disrupting the replication cycle of *Legionella*

*Legionella* have a biphasic lifestyle, and are found inside a broad range of protozoan hosts and in the extracellular environment (mostly embedded in biofilms) (Boamah et al. 2017, Mondino et al. 2020). However, there is common agreement that the replication of *Legionella* occurs predominantly inside protozoa, where *Legionella* use more than 300 effectors with considerable functional overlap to hijack the degradation mechanisms of the host cell and establish a *Legionella-Containing-Vacuole* (LCV) (Isberg et al.^2009^). From the LCV, *Legionella* recruit all the molecules and complexes they need in order to acquire nutrients and trigger the replicative phase (Bruckert and Abu Kwaik 2015, Prashar and Terebiznik 2015). Thus, disrupting this replicative pathway would present a major breakthrough in *Legionella* control.

*König* and colleagues showed that amoeba that harbour the symbiont *Protochlamydia amoebophila* survive infection by *Legionella pneumophila* and, importantly, that the numbers of intracellular *Legionella* were decreased compared to infections without *P. amoebophila*(König et al. 2019). Because the uptake of *Legionella* was not impaired, it is possible that nutrient competition drives this phenomenon, even though other possibilities (including the use of antimicrobial molecules produced by *P. amoebophila*) are still plausible (König et al. 2019). Other work has demonstrated that *Legionella* uptake can be prevented in *Amoeba* infected with the obligate intracellular symbiont *Neochlamydia*eS13 (Maita et al.^2018^). Although the mechanism is still unclear, it is suggested that the symbiont impairs the utilisation of the phagocytic pathway by *Legionella*. This is suggested to be a *Legionella* specific mechanism, since co-cultivations of *Neochlamydia* do not decrease uptake of other *Amoeba*-infecting organisms (Maita et al. 2018). Thus, these two chlamydial endosymbionts of *Acanthamoeba spp*. (*Protochlamydia amoebophila* and *Neochlamydia*) are able to provide protection for the host and inhibit *Legionella* growth, presumably through different modes of actions (competition and uptake inhibition).

### Possible *Legionella* grazing by some protozoa

Although *Legionella* infect many eukaryotic hosts and replicate intracellularly, studies showed that certain hosts can either avoid uptake and/or inhibit replication, or even actively graze on *Legionella*(Amaro et al.^2015^, Dey et al.^2009^, Rowbotham^1986^). Some of these interactions are influenced by external factors like temperature. For example, some natural host *Acanthamoeba* strains apparently graze and consume *Legionella* at temperatures lower than 25°C (Boamah et al. 2017, Ohno et al.^2008^). Also, some eukaryotic hosts apparently graze naturally on *Legionella*, irrespective of temperature. Amaro and colleagues (Amaro et al. 2015) identified and isolated protists whose abundance increased with the addition of *Legionella* in microcosm experiments, assuming that this phenomenon was determined by feeding on *Legionella*. They later confirmed that the protists *Solumitrus palustris, Paracercomonas CWPL*, and *Cercomonas MG33* were able to consume *Legionella* as a source of nutrition, as demonstrated by TEM and real-time PCR (Amaro et al. 2015).

In addition to this, two studies have demonstrated that the waterborne amoeba *Willaertia magna* is able to interfere with the mechanisms by which some strains of *L. pneumophila* exploit the intracellular environment in order to replicate (Dey et al. 2009, Hasni et al. 2020). In particular, when compared to the well-known *Legionella* hosts *Acanthamoeba castellanii* and *Vermamoeba vermiformis*, poor or absent intracellular proliferation was observed after the infection. Moreover, *W. magna* appeared to be highly resistant to *Legionella*-induced cell lysis, but it is also not conclusive that *W. magna* actually lyse *Legionella* (opposed to not facilitating replication) (Dey et al. 2009). Although the exact mechanisms that determine the resistance to the opportunistic pathogen are not yet clear, it is possible that the effectors normally used by *Legionella* to replicate inside the host might not work against *W. magna*, suggesting that *Legionella* are able to apply specificity in host selection and intracellular infection, as already discussed in other studies (Boamah et al. 2017). The possibility of using *W. magna* as a biological control strategy against *Legionella* is currently being explored by the French company *Amoéba* ((https://amoeba-nature.com/en/), although independent scientific evidence of the efficacy of this approach remains limited. Another recent finding identified two *Paramecium* strains (*P. multimicronucleatum Y-2 and P. multimicronucleatum YM-25*) in which *Legionella* were not able to establish intracellular replication (Watanabe et al. 2020). The two strains were demonstrated to digest intracellular *Legionella pneumophila Ofk 308* and *Philadelphia-1*, significantly reducing *Legionella*, although the mechanisms leading to this elimination also remain to be elucidated.

### Research gaps and future perspectives

An approach focusing on the protozoan hosts offers research opportunities in two key areas, namely (i) exploiting bacterial symbionts, which directly interfere with the intracellular replication of *Legionella*, and (ii) identifying and characterizing more protozoa that actively grazes on *Legionella* and/or impair their replication. With respect to the first, proof-of-principle research should focus on identifying non-pathogenic protozoan symbionts, and then performing co-infection assays for selecting suitable *Legionella* inhibitor strains, while more knowledge is needed on the specificity, and this broader applicability of such a strategy. For the second approach, there are limited detailed descriptive studies of the eukaryotic drinking water microbiome. Several studies have however reported evidence of protists grazing on *Legionella*, and it is therefore likely that drinking water harbours more non-permissive eukaryotes able to prevent the infection of *Legionella*. Thus, a next step would be identifying and isolating more potential grazing protozoa, screening different hosts in co-culture experiments with *Legionella*, characterising the mechanisms involved in *Legionella* inhibition, and eventually testing the approach in more realistic microcosm environments. While the second strategy appears to be promising, the possibility that *Legionella* could evolve to infect these non-permissive hosts has to be considered as a potential problem. *Legionella* can in fact grow in a vast range of eukaryotes (Boamah et al. 2017). This evolutionary advantage has been acquired through the progressive expansion of the genome under selective pressure to allow the bacterium to replicate inside of a range of different hosts (O'Connor et al.^2011^).

## Competition with other bacteria

### Molecular evidence exists for competition within the drinking water microbiome

Bacteria continuously compete with each other for resources, producing negative fitness consequences for the recipient whilst benefiting the actor (Granato et al.^2019^). In ecology, competition is defined in two categories, exploitative competition (indirect, and occurs when resources are consumed by some organisms with negative consequences for others) and interference competition (direct, and occurs when an organism is negatively affected by the action of another organism with modalities that interfere with their growth, but do not involve increased nutrient uptake in one of the competitors (Granato et al. 2019)).

Several studies have reported (possible) antagonistic relationships between *Legionella* and other bacteria in engineered aquatic environments. For example, *Pseudomonas* has been identified as a genus that is enriched when *Legionella* was repressed or absent in cooling towers (Paranjape et al. 2020), drinking water shower hoses (Proctor et al.^2018^), and swimming pools (Leoni et al. 2001). Other taxa have sporadically been high in abundance when *Legionella* were absent, including *Sphingobium* in cooling towers (Paranjape et al. 2020) and *Bacteroidia* and *Solibacteres* in high-rise building water (Ma et al. 2020). However, it is important to note that these observations do not identify specific competition pathways or demonstrate causation to the observed correlations. Nevertheless, they suggest that competitive anti-*Legionella* interactions may occur in engineered aquatic environments and give first guidance to focus on specific taxa that may be exploited to develop anti-*Legionella* control strategies. Below we discuss documented examples of interference competition between *Legionella* and other bacteria, and explore possibilities for exploitative competition for nutrients that would take advantage of the specific nutrient requirements of *Legionella*, proposing mechanisms that could justify a future probiotic approach.

### Interference competition: antimicrobial compounds against *Legionella*

All major bacterial phyla can produce toxic compounds such as antibiotics and bacteriocins in order to kill or inhibit competitors. These interactions can either be contact-dependent (via e.g., injection of toxic proteins into neighbouring cells), or contact-independent, where toxins diffuse freely in the environment (Hibbing et al. 2010, Peterson et al.^2020^, Riley and Gordon 1999). The first reported strain to produce two bacteriocin-like-proteins able to inhibit the growth of *Legionella* was *Staphylococcus warneri*, which exhibited an inhibition zone of *Legionella* growth in a spot-on-lawn assay (Hechard et al.^2005^). Three molecules produced by *S. warneri* (warnericin RK, delta-lysin I, and delta-lysin II) were subsequently identified as the causal agents for the *Legionella* inhibition (Verdon et al.^2008^). Because the molecules have comparable physical-chemical properties (e.g., tridimensional structures), they are thought to have similar modes-of-action. Another bacterial antimicrobial compound is surfactin, produced by *Bacillus subtilis* AM1. Surfactin acts against *Legionella* at very low concentrations, decreases levels of host organism *Acanthamoeba castellanii* at high concentrations, and was demonstrated to disperse a pre-formed *Legionella* biofilm (Loiseau et al. 2015). The same authors demonstrated that rhamnolipid and lipopeptide biosurfactants produced by members of the *Pseudomonas* genus are also able to inhibit *Legionella* (Loiseau et al. 2018). Since all the molecules mentioned above interact with the cell membrane, this suggests that some features on the outer membrane might be the cause for this antimicrobial activity. Furthermore, one recent study identified the virulence factor toxoflavin as a compound produced by *Pseudomonas alcaliphila* with inhibitory activity towards *Legionella pneumophila* and their host *Vermamoeba vermiformis*(Faucher et al.^2022^).

Other researchers examined whether bacteria that produce bacteriocins or bacteriocin-like substances (BLSs), proteinaceous molecules that have a killing activity on strains belonging to the same or closely related species, could inhibit *Legionella* (Guerrieri et al. 2008). These authors showed that 69% of 80 BLS-producing bacterial strains exhibited inhibition against *Legionella*. Of the 11 species or taxa tested, all except *Acinetobacter* spp. exhibited *Legionella* inhibition in at least 50% of the isolates screened. However, other mechanisms of *Legionella* inhibition associated with these strains (e.g., production of other molecules rather than bacteriocins; nutrient competition, etc) cannot be ruled out. Corre and colleagues tested 273 isolates from five different environmental water sources for anti-*Legionella* activity using spot-on-lawn assays (Corre et al. 2018). The majority of the isolates inhibiting *Legionella* belonged to the genus *Pseudomonas*, but also *Flavobacterium* spp*., Aeromonas* spp*., Bacillus, Chryseobacterium* spp*., Kluyvera* spp., and *Ralstonia* spp.. These authors furthermore hypothesised that the production of volatile compounds capable of killing *Legionella* was responsible for *Legionella* inhibition, based on long-range-inhibition assays performed in multi-well plates, and later identified 1-undecene as the main volatile compound responsible for the antagonistic activity (Corre et al. 2021).

### Potential exploitative competition for growth limiting nutrients

*Legionella* require iron to replicate, infect host cells, and are auxotrophic for several amino acids (Byrd and Horwitz 2000, Chien et al. 2004, Cianciotto 2007, Gebran et al. 1994, George et al.^1980^, James et al.^1995^, Reeves et al.^1981^, Tesh and Miller 1981). In particular, cysteine and serine appear most important (Ewann and Hoffman 2006; Eylert et al. 2010). These rather specific nutrient requirements present opportunities for exploitative competition strategies to limit *Legionella* growth.

Many bacteria produce molecules called siderophores, which use receptors that are specific to the organism producing them, to help solubilize and transport iron to the cell (Hider and Kong 2010). This may enable competition with *Legionella* for iron either by producing different types of siderophores or by “stealing” *Legionella*- produced siderophores (Figueiredo et al.^2021^, Niehus et al.^2017^). Exploiting siderophores production of some bacteria (e.g., *Bacillus* spp., and *Vibrio* spp.) has been used with different applications in other fields, such as medicine, plant biology, biocontrol of fish pathogens, bioremediation (Ahmed and Holmström 2014, Kurth et al. 2016).The bacterium *Pseudomonas aeruginosa* can modulate the production of siderophores and even produce different types of siderophores under competition and when iron is limited (Leinweber et al.^2018^). The fact that *Pseudomonas* were previously documented to have negative correlations with *Legionella* (above), suggests that siderophores production for iron competition could be one mechanism contributing to these observational studies. While *Legionella* are also able to produce a type of siderophores, legiobactin (Liles et al.^2020^), the competitive nature of these molecules, as well as the fact that they are widespread among different bacterial species, might put *Legionella* in disadvantage compared to the other members of the community, especially if the siderophores produces by them are more affine to iron and/or their production can be regulated.

Competitive growth of microorganisms that have a higher affinity for the amino acids that are the primary carbon source for *Legionella* represent another potential mechanism to create detrimental nutrient limitation for *Legionella*. The system commonly used by bacteria to take amino-acids up is represented by ABC transporters, which in some cases are specific for one amino acid and in other cases can promote the internalization of a broad range of amino-acids with various overlap (Hosie and Poole 2001). So far, however, substantial information is lacking on the way bacteria (and specifically *Legionella*) compete for amino acids, and if and how the above-mentioned transporters are involved in these competitive dynamics.

### Research gaps and future perspectives

The competitive mechanisms discussed above predominantly target *Legionella* survival and replication in biofilms outside of their protozoan hosts. Given that this is not considered the primary replication pathway of *Legionella*, the efficacy of any eventual probiotic strategy based on competitive interactions may be limited. When inside of protozoa, *Legionella* acquire nutrients from the intracellular environment through different effectors that facilitate the uptake of essential amino-acids and iron in the *Legionella*-containing vacuole, thus rendering any strategies based on competition for nutrients ineffective (Isaac et al.^2015^, Richards et al.^2013^). However, the fact that many diverse antagonistic organisms have been isolated and demonstrated to inhibit *Legionella* growth through direct interference competition on agar is promising, especially as a complementary strategy targeting *Legionella* growing/surviving outside of their hosts Further studies could expand the list of anti-*Legionella* strains and identify the molecules and mechanisms involved. Moreover, previous studies were mostly done in artificial environments, and considerably more research, specifically under representative conditions for specific engineered environments, are needed for such an approach to become realistic. In terms of exploitative nutrient competition, a first probiotic approach should probably focus on bacteria with a high iron affinity and ideally able to produce a range of iron-scavenging siderophores able to trigger competition with *Legionella*, as well as microorganisms with similar amino-acids requirements. One potential more efficient way to exploit interference and nutrient competition would be identifying competitive mechanisms towards the host itself, with resulting negative consequences for intracellular *Legionella* replication. While highly interesting, this entire topic lags considerably behind others with respect to basic research demonstrating the underlying concepts.

## Predation as a means to control *Legionella* in aquatic systems

### Predatory bacteria

Predatory bacteria are a diverse phylogenetic group that actively kill their bacterial prey and absorb the prey's macromolecules as nutrients (Pérez et al.^2016^, Sockett and Lambert 2004). This includes organisms such as *Micavibrio aeruginosavorus*(Wang et al. 2011), *Bdellovibrio exovorus* (Koval et al. 2013), *Bdellovibrio bacteriovorus* (sockett and Lambert2004) and *Myxococcus xanthus* (Keane and Berleman 2016). The predatory bacterium *B. bacteriovorus* has previously been proposed as a probiotic and ‘living antibiotic’ (Dwidar et al. 2012, Sockett and Lambert 2004, Tyson and Sockett 2017), with examples including biocontrol of fish and shellfish pathogens (Cao et al.^2015^, Cao et al. 2014, Chu and Zhu 2010) and control of clinical pathogens including multidrug-resistant ones (Atterbury and Tyson 2021, Negus et al. 2017, Sockett and Lambert 2004).

To date, only two studies investigated whether *B. bacteriovorus* has the ability to lyse *Legionella*, both under laboratory conditions. Markelova (Markelova 2010) found that *Legionella* L100 was not susceptible to *B. bacteriovorus* HD100 predation. In contrast, an earlier study of Tomov et al. (Tomov et al. 1982) showed that *Legionella micdadei* (strain Tatlock), *Legionella bozemanii* (strain Wiga) and *L. pneumophila* (several strains belonging to serogroup 1, 2, 3, or 4), were lysed by *B. bacteriovorus* strains 6-5-S and 12. This latter study suggests some future potential for this approach. When evaluating feasibility of *B. bacteriovorus* as a predatory biocontrol agent against *L. pneumophila* in engineered aquatic ecosystems, multiple aspects have to be taken into consideration. First is the efficacy in reducing multispecies biofilms, as this is a primary niche for *Legionella* (Declerck 2010). In general, *B. bacteriovorus* has shown promising efficacy to prevent and reduce biofilms colonized by a wide range of Gram-negative pathogens in a laboratory setting (Dashiff et al. 2011, Kadouri and O'Toole 2005, Sun et al. 2017). However, up to now there are no evidences of specific features rendering *Legionella* a more or less specific target for predatory bacteria, and it is unknown whether predation can be effective in multi-species biofilms under realistic conditions and/or if altering the drinking water microbiome might have unintended consequences (e.g., removal of beneficial species). Secondly, because *Legionella* survive and proliferate in a wide variety of protozoa, and since *B. bacteriovorus* typically attacks free *L. pneumophila* cells, this may limit effective application of a predatory approach. However, disrupting the balance between free-living *Legionella* and amoeba-associated *Legionella* may already disrupt the overall *Legionella* life cycle in a system. Moreover, target-specificity is a factor to be considered. While predatory bacteria are not extreme generalists nor specialists with respect to their target organisms (Johnke et al. 2014), an ideal probiotic strategy would target as many pathogenic *Legionella* spp. as possible. Because different *Bdellovibrio* spp. can have a different (i.e. broader) prey range (Jurkevitch et al.^2000^), the identification of one or more suitable strains capable of targeting a large number of pathogenic *Legionella* spp. would be a priority. Finally, the safety of any probiotic culture has to be considered. Many *Bdellovibrio* species are present in natural and engineered aquatic systems, including mains water supplies (Richardson, 1990), shower hoses (Neu et al. 2019, Proctor et al. 2016), and wastewater treatment plants (Feng et al. 2016, Fry and Staples 1976). Encouragingly, no adverse health impacts of *B. bacteriovorus* on animals by oral administration and respiratory inoculation have been found (Atterbury et al. 2011, Shatzkes et al. 2015, Shatzkes et al. 2016). The latter point is particularly important, given *Legionella* transmission requires aerosolization of the bacteria (Fields et al. 2002, Mondino et al. 2020).

### Phage therapy

Bacteriophages are viruses that infect bacteria. They are often very selective and infect only a certain species (or sometimes even specific strains) of bacteria. Phages can be either lytic or lysogenic. Lytic phages infect their host, replicate, and burst their host open to find new host bacteria to infect. Hence, only lytic phages are used for phage treatment. Phages have previously been proposed as alternative antimicrobial strategies, with numerous examples in human-associated-pathogen control (De Paepe and Petit 2014), biofilm control (Donlan 2009, Motlagh et al. 2016, Parasion et al.^2014^), and in the food sector (Fernandez et al. 2018). In a recent relevant example, researchers isolated phages against the opportunistic pathogen *Pseudomonas aeruginosa*, as a first step toward alternative remediation strategies for contaminated water systems (Kauppinen et al. 2021).

To date, no phages that target *Legionella* have been confirmed. Lammertyn and colleagues claimed to have isolated four phages of the Myoviridae family able to infect *Legionella* strains (Lammertyn et al. 2008). In the same year, Grigor'ev and colleagues (Grigor'ev et al. 2008) claimed to have isolated a temperate phage from a guinea pig infected with *L. pneumophila*. Other studies have struggled to isolate any *Legionella* phages (e.g., PhD thesis, (Nezam-Abadi 2019)). To our knowledge, no further *Legionella* phages have been isolated after the initial 2008 studies, and no enrichments exist of the previously described isolates. Despite the lack of isolates, there is some genetic evidence for *Legionella* phages. For example, Gomez-Valero and colleagues claimed identification of the first complete prophage in the genome of *L. micdadei* and another study of the *Legionella* genome concluded that *L. pneumophila* phages are most likely lytic gokushoviruses (Deecker et al.^2021^, Gomez-Valero et al. 2014). Both of the aforementioned studies argued that *Legionella* phages are probably very rare. The reason for this might be related to the intracellular lifestyle of *Legionella*, which could in some way protect the pathogen against phages (Rao et al. 2016). However, the presence of a CRISPR-Cas system in several *Legionella* strains has been reported, indicating that those might have previously been exposed to bacteriophages (D'Auria et al.^2010^, Deecker et al. 2021, Faucher and Shuman 2011, Rao et al. 2016). While this could represent a reason to continue investigating potential phages targeting *Legionella*, it also creates a problem for the application of a phage-based probiotic approach, since a CRISPR-Cas system provides bacteria with protection against the reinfection of the same phage (Deveau et al.^2010^).

### Research gaps and future perspectives

Predatory control of *Legionella* seems to hold interesting research possibilities, but at the same time remains insufficiently investigated. Further studies are necessary prior to a potential specific application of predatory bacteria in a engineered water system that considers the biofilm and amoeba facets of *Legionella*’s life cycle. Important is also the assessment of any eventual resistance that *Legionella* might develop towards predatory bacteria, for which further research would be needed. With respect to phages therapy, we can conclude that any potential treatment against *Legionella* remains at this stage only a scientific pipe dream. It is evident that considerable additional efforts would be needed to first isolate, characterise and enrich *Legionella* phages, since that success in this regard has to date been extremely limited.

## Considerations for practical feasibility and public acceptance of potential probiotic strategies

### Feasibility

It is evident from the information summarised above that a considerable body of research is needed to develop a probiotic approach into actual prevention or treatment strategies. Any strategy would require organisms that (i) are completely benign, (ii) can survive (and ideally establish) in diverse engineered water systems, (iii) effectively inhibit *Legionella* proliferation, and (iv) do not alter the microbial environment negatively in the long run. A probiotic approach would require at least some integration in the overall drinking water system, which also means that regional differences in the chemical properties of drinking water should be considered. For example, heavily chlorinated drinking water may negatively impact certain probiotic strategies, leading to inconsistent outcomes. In contrast, non-chlorinated drinking water (e.g., Switzerland, Denmark) comprise complex indigenous communities that would provide considerable competition for probiotic strains. Any probiotic strategy would most probably need to be used in combination with an existing strategy (e.g., improving temperature circulation in hot water systems) and may require a consortium of probiotic organisms with functional redundancy and/or diversity since any one-probiotic strain may not effectively colonize a given aquatic ecosystem. Moreover, in order to function at its best, a probiotic community would have to outcompete part of the resident microbiota in a given system. This could potentially be achieved through pre-colonisation of new systems with the probiotic community, but it would probably be problematic to achieve in older systems with established microbial communities. For the latter, a potential approach maybe a combination with harsh pre-treatments (i.e. high concentration chemicals) to get rid of the resident microorganisms, prior to the repopulation with probiotic species. While any probiotic organism must be harmless, they should also not promote the growth of different opportunistic pathogens. Further, a one-time treatment may not be effective; as with other engineering controls, remedial efforts may require re-application or continual application for success. The drinking water industry is historically fraught with unintended consequences, and unintentional selection for other opportunistic pathogens must be considered and avoided (e.g., increased numbers of *Mycobacterium avium* after the use of chloramines to treat *Legionella* (Rhoads et al.^2017^)).

The fundamental research that aims to identify individual probiotic microorganisms is only the first of many next steps to assess the feasibility of any probiotic approach. Any probiotic must be tested against diverse *Legionella* strains, focusing on the ones that are known to be responsible for the majority of disease in controlled artificial conditions before being tested in more realistic conditions (microcosms, biofilms). One possible approach to move from lab- to full-scale research is to pilot-test the approaches in a given system before application, analogous to optimized corrosion control studies in water distribution systems, providing information on how a cross-section of probiotic approaches may perform in a given system to select the best one(s). If successful probiotics can be defined, the final challenge would be to design and validate a monitoring strategy to ensure their long-term efficacy. We believe that amoeba-related probiotic strategies hold the highest promise among all strategies evaluated in this article, given the host-pathogen relationship is critical to *Legionella* survival and replication in drinking water systems.

### Public acceptance

While the safety and efficacy of any probiotic or predatory biocontrol strategy are the top priorities in defining potential solutions, public acceptance must also be considered. Application of promising approaches may benefit from past efforts such as modulation of the gut microbiome to treat enteric disease (Baunwall et al. 2021), phage therapy in the food industry to prevent foodborne illness (Endersen and Coffey 2020), and addressing the “yuck” factor associated with water reuse efforts (Fielding et al.^2018^). Thus, effective scientific communication would be needed to clarify that probiotics would be intentionally shaping the microbes in a system, not adding them to a “pure” system. One particular challenge to overcome is that public acceptance might be easier for other probiotics that are administered on an individual level, while a probiotic approach for a shared system such as building plumbing would affect all consumers in a given system. However, the public already generally accepts centralized water treatment that supplies non-chlorinated, chlorinated, or chloraminated water to communities, each with their own set of implications for public health. In addition, probiotic-based-treatments may not necessarily need to be applied at a general level (as for chemicals), but might be used as targeted approach for specific contaminated buildings. Moreover, probiotic strategies may well be better accepted if first tested and demonstrated on engineered aquatic ecosystems without direct public water consumption/exposure (i.e. cooling towers).

## Conclusion

- A probiotic approach to control *Legionella* should take advantage of its interactions with the surrounding microbial community at multiple trophic levels, possibly in combination with other treatments (e.g., thermal or chemical shock), using multiple complementary strategies with redundant or diverse modes of action.
- We believe that interrupting the host-pathogen relationship with protists is the most feasible long-term approach, but competition and predation may still support probiotic success at the start of probiotic treatment.
- Fundamental research to identify more probiotic bacterial and protozoan strains with functional redundancy and diversity through bench-scale screening assays is a logical next step towards assembling promising candidates of probiotic approaches.
- Feasibility testing in realistic engineered aquatic systems and public acceptance should also be considered while developing the body of fundamental research needed to design successful probiotic strategies.
